# Expression of Endonuclease *Rsa*I Induces Chromosomal Rearrangement in the Yeast *Kluyveromyces marxianus*

**DOI:** 10.3390/cimb48030252

**Published:** 2026-02-26

**Authors:** Babiker M. A. Abdel-Banat, Muhammad Munir, Hisashi Hoshida, Rinji Akada

**Affiliations:** 1 Date Palm Research Center of Excellence, King Faisal University, Al-Ahsa 31982, Saudi Arabia; 2 Division of Applied Chemistry, Graduate School of Science and Technology for Innovation, Yamaguchi University, 2-16-1 Tokiwadai, Ube 755-8611, Japan; 3 Research Center for Thermotolerant Microbial Resources, Yamaguchi University, 1677-1 Yoshida, Yamaguchi 753-8315, Japan

**Keywords:** chromosomal rearrangement, DSB repair, genome instability, *Kluyveromyces marxianus*, NHEJ, *Rsa*I endonuclease

## Abstract

DNA double-strand breaks (DSBs) are primarily repaired in eukaryotic cells through two pathways: homologous recombination (HR) and non-homologous end joining (NHEJ). The thermotolerant yeast *Kluyveromyces marxianus* is recognized for its highly active NHEJ pathway, making it a suitable model organism for studying the role of NHEJ in DSB repair. To induce DSBs in *K. marxianus* DMKU3-1042, an expression cassette containing the gene encoding the endonuclease *Rsa*I was integrated into the *LYS1* locus of both the wild-type and NHEJ-deficient *KU70* mutant strains. This cassette is regulated by the galactose-inducible promoter *GAL10*. Cells expressing *Rsa*I and grown in galactose medium exhibited an elongated, rod-shaped morphology under a microscope. Following *Rsa*I expression, the viability of transformed *KU70* cells decreased during the first three hours of culture in liquid medium and then partially recovered after six hours of incubation. In contrast, the *KU70* mutant cells failed to produce viable survivors. Pulsed-field gel electrophoresis analysis revealed distinct chromosomal separation patterns among various *Rsa*I-transformed *KU70* cells. These findings demonstrate that the repair of *Rsa*I-induced DSBs in *K. marxianus* DMKU3-1042 results in new strains with several forms of rearranged chromosomes.

## 1. Introduction

Genomic alterations in yeast cells can occur spontaneously or be induced by external factors [1]. Such alterations include point mutations, chromosomal rearrangements (such as large deletions, duplications, inversions, and translocations), and whole-chromosome aneuploidy. Structural chromosomal rearrangements are alterations in chromosome structure that involve the exchange of large segments of DNA. These rearrangements can have significant effects on gene function and genome stability. The primary mechanisms underlying structural chromosomal rearrangements include DNA double-strand breaks (DSBs), defective DNA repair mechanisms, aberrant recombination events, and chromothripsis [2,3], a mutational pattern in which a large number of rearrangements are confined to local regions, often accompanied by copy-number losses [4]. These chromosomal fragments are then incorrectly reassembled simultaneously, rather than accumulating chromosomal rearrangements gradually over many generations [3]. This process is driven by errors during mitosis, which generate abnormal nuclear structures that lead to extensive but localized fragmentation of mis-segregated chromosomes [5]. These structural chromosomal rearrangements are often associated with specific genomic architectural features that can lead to genetic instability [6]. DSBs are among the most detrimental types of DNA damage, leading to genome instability, apoptosis, or cell senescence if not correctly repaired [7,8,9,10,11,12]. Nevertheless, in some instances, these genomic rearrangements can yield mutant strains capable of growing under highly acidic conditions (pH 1.8) in the non-conventional yeast *Candida utilis*, via direct nuclease transfection [13].

In eukaryotes, DNA repair primarily occurs through non-homologous end joining (NHEJ) or homologous recombination (HR) to maintain genome integrity [14]. The process proceeds through a cascade of events in which DNA damage sensors, transducers, and effectors detect and rejoin the broken ends of chromosomes [14,15]. HR depends on a considerable homology between the damaged DNA and an undamaged partner on a sister chromatid or a homologous chromosome [16,17]. HR requires proteins of the modifier gene group *RAD52* (*RAD52*, *RAD51*, *RAD54*, and *RAD55*/*57*), in addition to several nucleases and helicases [18,19,20]. Moreover, the NHEJ repair pathway requires little or no homology between the joining DNA [14] and the proteins Ku70, Ku80, and Lig4 [21].

Defects in the repair of conserved DNA pathways may lead to cell death or chromosomal rearrangements such as translocations, duplications, deletions, inversions, and the formation of rearranged chromosomes [22,23,24]. Extrachromosomal circular DNA (eccDNA) is one form of chromosomal rearrangement found in cancer genomes and multiple genetic diseases [23,25,26]. Chromosomal rearrangements also play a role in phenotypic divergence and environmental adaptation, as evidenced in some organisms such as *Plasmodium falciparum* [27], *Candida albicans* [28], *Drosophila* S2 cells [29], and *Saccharomyces cerevisiae* [30].

The consequences of DSB repair have been extensively studied in model organisms such as *S. cerevisiae* [31]; however, limited information is available on how these processes impact the chromosomal integrity of *Kluyveromyces marxianus* (*Km*). The main advantage of *K. marxianus* is its robust NHEJ activity compared to the yeast *S. cerevisiae* [32,33]. Using this characteristic, *K. marxianus* randomly integrates linear DNA into its chromosomes. This feature enabled the identification of auxotrophic mutant genes, obviating plasmid construction in *Escherichia coli* [32,33]. In addition, this yeast can generate circular plasmid DNA when the heterologous DNA contains the innate autonomously replicating sequence (*Km*ARS), which aided in constructing plasmids harboring various selection markers and recombinant DNAs [34,35]. A study on *Km*ARS point mutations, deletions, and nucleotide substitutions was also performed using this robust NHEJ activity in *K. marxianus*, which resulted in the identification of the smallest sequence that functions as an ARS in *K. marxianus*. The sequences from different *Km*ARSs were found to be interchangeable, enabling robust autonomous replication [36]. Using CRISPR-Cas9-induced chromosome breaks, a multigene integration tool was developed in *K. marxianus* via its highly active NHEJ pathway, resulting in enhanced 2-phenylethanol biosynthesis [37]. These characteristics of *K. marxianus* suggest that this yeast is a suitable model organism for investigating the role of the NHEJ pathway in the repair outcomes of DSBs.

In this study, we aim to explore the effect of induction of the blunt-end restriction enzyme *Rsa*I that recognizes the GT/AC sequence and whether the induced widespread DSBs could be repaired by the highly active NHEJ pathway in *K. marxianus* to generate diverse and viable chromosomal rearrangements, in addition to determining whether this process will be impaired in a *ku70Δ* background.

## 2. Materials and Methods

### 2.1. Strains and Culture Media

The yeast strains used in this study are listed in Table 1. YPD medium (1% yeast extract, 2% peptone, and 2% glucose) and minimal medium (MM; 0.17% yeast nitrogen base without amino acids and ammonium sulfate, 0.5% ammonium sulfate, and 2% glucose) were prepared following a previously described method [38]. YPGal medium contains 1% yeast extract, 2% polypeptone, and 4% galactose. The dropout MM-U contains [adenine sulfate, L-tryptophan, L-histidine HCl, L-methionine, L-leucine, and L-lysine HCl] and lacks uracil [39]. MM-Leu and MM-Lys are dropout MM-U media that lack L-leucine and L-lysine, respectively. To prepare a 5-fluoroorotic acid (FOA) medium, 50 mg/L of uracil and 2% agar were added to the MM-U (MM-U + uracil) medium, autoclaved, and subsequently maintained at 65 °C in a water bath. FOA was dissolved in dimethyl sulfoxide (DMSO) at 100 mg/mL and then added to autoclaved MM-U + uracil medium to achieve a final FOA concentration of 1 mg/mL [40]. For the preparation of solid plates, 2% agar was added.

### 2.2. Construction of Yeast Strains Expressing RsaI

A construct containing the gene for the restriction enzyme *Rsa*I was designed for expression in *K. marxianus* under the control of the *GAL10* promoter (P*_GAL10_*). The activity of this promoter is tightly regulated by the induction of galactose in *K. marxianus*. The yeast *S. cerevisiae* strain RAK3940 (Table 1) was used as a transformation host to replace the construct P*_GAL10_*-yEGFP-15C-PpHIS3. *S. cerevisiae* strain RAK5331 with a fusion construct of P*_GAL10_*-*Rsa*I-*URA3* was obtained. The fusion construct P*_GAL10_*-*Rsa*I-*URA3* was amplified from the *S. cerevisiae* strain RAK5331 using the previously described fusion PCR method [42,44]. *KmLYS1* homologous sequences (1 kb) were attached to both ends of the fusion construct [*KmLYS1*-P*_GAL10_*-*Rsa*I-*URA3*-*KmLYS1*] and amplified using the primers KmLYS1URA3-1000: (5′-AATGGCTCCCACATTGCTGTTGGGTTATTG-3′) and KmLYS1URA3-1000c: (5′-AGATTGATATATCTAGTACTACCGAAA-3′). Thereafter, the fusion construct was targeted at the *LYS1* locus of the *Km* Ku70^+^ and Km Ku70^−^ strains using the *Km*-targeted gene integration method [33,45], as explained below in the section “2.3 *Km* transformation”. The resulting transformant strains were RAK5551, which retained the intact *KmKU70* gene, and RAK5332, which lacked the *KmKU70* gene (Table 1). The *K. marxianus ACT1* gene was amplified as a reference gene for PCR amplification using primers KmACT1 + 16 (5′-GCAGAGGTCGCTGCTTTAGTTATTG-3′) and KmACT + 1111c (5′-ATGGACCAGATTCGTCGTATTCTTG-3′). The sequence of the fusion construct [*KmLYS1*-P*_GAL10_*-*Rsa*I-*URA3*-*KmLYS1*] is shown in Supplementary Figure S1.

### 2.3. Km Transformation

The transformation mixture (TM) consists of the following components in final concentrations in sterilized distilled water: 40% *w/v* polyethylene glycol 3350 (PEG), 200 mM lithium acetate (LiAc), and 100 mM dithiothreitol (DTT). Single-stranded carrier DNA was added to the transformation reaction at a final concentration of 1 mg/mL in TM [33]. *K. marxianus* cells were grown overnight in YPD, diluted 1:10 in 18 mL of fresh YPD, and allowed to grow for 5 h at 28 °C with vigorous shaking. The cells at the log phase were centrifuged, washed once with 500 μL of TM, and immediately suspended in 180 μL of TM. The cell suspension (85 μL) was transferred into a new 1.5 mL tube and mixed with 10 μL of 10 mg/mL denatured carrier DNA and 5 μL of purified PCR-fusion DNA (*KmLYS1*-P*_GAL10_*-*Rsa*I-*URA3*-*KmLYS1*). The *URA3* gene from *S. cerevisiae* (*ScURA3*) with flanking 1 kb *KmLYS1* homologous sequences (*KmLYS1*-*URA3*-*KmLYS1*) was also transformed as a control. The mixture was vortexed for 30 seconds, incubated at 42 °C for 40 min, and then spread onto the required selection/dropout plates. The plates were incubated at 28 °C for 2–3 days.

### 2.4. Measurement of Yeast Viability Under RsaI Restriction Enzyme Expression

The wild-type strain DMKU3-1042 of *K. marxianus* and the strains expressing the restriction enzyme *Rsa*I (RAK5551 and RAK5332) were cultured in deep-bottomed Petri dishes containing 5 mL of YPD and incubated for 18 h at 28 °C with vigorous shaking at 150 rpm. All strains exhibit normal growth under the non-inductive medium (YPD). The yeast cultures were transferred to 15 mL Falcon tubes and centrifuged at 6400 RCF for 3 min, and the supernatant was discarded. The cell pellets were washed twice with 5 mL of sterile deionized water (SDW) and resuspended in 5 mL of SDW. The suspended cells’ optical density (OD_600_) was measured using a WPA CO 8000 cell density meter (Biochrom Ltd., Cambridge, UK). The samples were prepared in a 15 mL tube by diluting them with sterilized deionized water to achieve an optical density (OD_600_) of 2. Five milliliters from each of the three strains was added to Erlenmeyer flasks containing 2× YPD medium, resulting in a total volume of 10 mL. Similarly, 5 mL from each of the three strains was added to flasks containing 5 mL of 2× YPGal medium, resulting in a total volume of 10 mL. The flasks were incubated in a rotary shaker at 37 °C with vigorous shaking. Samples were taken at 3 h intervals. On each occasion, 100 μL of the sample was transferred into a chilled 1.5 mL tube containing 900 μL of sterile deionized water. DMKU3-1042, RAK5551, and RAK5332 strains cultured in YPGal medium were diluted to the same concentrations. An aliquot of 300 μL from each sample was plated onto YPD solid medium. The YPD plates were subsequently incubated for two days at 28 °C.

The colonies that had grown on the YPD solid medium were counted. The percentages of viable RAK5551 and RAK5332 strains over time were calculated as units per milliliter for each time point, after culturing the strains in YPGal medium with vigorous shaking. Three biological replicates were conducted for each test.

### 2.5. Microscopic Observation of Cells

The strain RAK5551 was cultured in YPGal with vigorous shaking for 6 h. An aliquot of 300 μL of the culture was plated on FOA-Gal to select for the loss of the *URA3*-*Rsa*I cassette. Recovered colonies were examined to assess the potential yeast chromosomal rearrangements. DMKU3-1042 and the recovered RAK5551 colonies were cultured in YPGal medium, grown at 30 °C for 24 h, and examined under a Nikon Eclipse TE2000-S inverted fluorescence microscope (Nikon Corporation, Tokyo, Japan) to observe cell morphology. Three independent microscopic observations were conducted for each strain.

### 2.6. Preparation of Yeast Chromosomal DNA in Agarose Inserts

Yeast cells were grown overnight in YPD, and final cell densities were estimated using a spectrophotometer. Yeast cultures were spun at 6400 RCF for 2 min. Cell pellets were washed twice in 50 mM EDTA (pH 7.5). Each time, the pellets were spun at 6400 RCF for 2 min, and the supernatant was discarded. The cell pellets were re-suspended in 50 mM EDTA buffer (pH 7.5) at 2× the cell concentration used for each insert. Three hundred microliters of the suspension was mixed with 6 μL of 2 mg/mL Zymolyase 100 T and 305 μL of 1.8% liquefied agarose (Low Melt Preparative Grade, Bio-Rad, Tokyo, Japan) prepared in 50 mM EDTA (pH 7.5) and equilibrated to 45~50 °C. The mixture was poured into plastic molds to enable the gel to cool and solidify. The gel inserts were removed from the mold and transferred to a 0.5 M EDTA (pH 7.5) solution containing 7.5% 2-mercaptoethanol (5 mL per 20 inserts). Inserts were incubated overnight at 37 °C without shaking. This treatment process removes cell wall material, leading to the formation of spheroplasts [46,47,48]. Thereafter, the inserts were incubated for 2 days in a solution containing 1% sodium lauroyl sarcosine as a detergent, 0.5 M EDTA (pH 9~9.5), and 1 mg/mL of proteinase K (this solution is referred to as ESP). A total volume of 5 mL of ESP was used per 20 inserts. The inserts were then stored at 4 °C indefinitely. Before performing pulsed-field gel electrophoresis (PFGE), the inserts were rinsed three times with TE50 buffer (10 mM Tris-HCl, pH 7.5, 50 mM EDTA, pH 7.5) to dilute the N-lauryl sarcosine [47,49].

### 2.7. PFGE for Chromosome Analysis

Electrophoretic separation of *K. marxianus* chromosomes in agarose inserts was performed according to standard protocols [48,50,51,52] with minor modification. Briefly, yeast chromosomes were separated using a chromosomal DNA electrophoretic apparatus, the BS-80, manufactured by Bio Craft (EverSeiko Corporation, Tokyo, Japan). The electrophoretic system consists of a Bio Craft model BE-900 electrophoresis tank (Cooling electrophoresis bath), connected to a Bio Craft pulse timer BC-950 and a Bio Craft real power model BP-4 power supply. The following conditions were used to separate *K. marxianus* chromosomes: the final agarose concentration was 0.9%, and the agarose was Certified^TM^ Molecular Biology Agarose (Bio-Rad, Tokyo, Japan). The pulse time was set at 150 s at a constant voltage of 140 V. The electrophoresis-running buffer was 0.5× TBE (89 mM Tris base, 89 mM boric acid, and 2 mM EDTA, pH 8.0). Electrophoresis was run for 35 hours at 14 °C. The PFGE experiment was repeated four times.

### 2.8. Statistical Analyses

Viability data for the strains were subjected to ANOVA (*p* < 0.05) using Statistix 8.1 software [53]. Comparison of means was performed using Tukey’s test at the 0.05 level of significance [54].

## 3. Results

### 3.1. Construction of K. marxianus Strains Expressing RsaI and Viability Measurement

**Figure 1:**
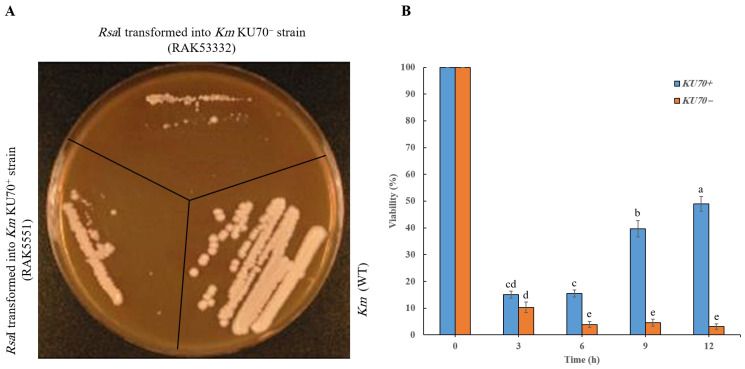
Growth properties of *Rsa*I-expressing *K. marxianus* transformants. (**A**) Growth of *KmKU70*^+^ and *KmKU70*^−^ strains expressing *Rsa*I on a static culture (YPGal plate) compared to the *Km* wild-type. (**B**) Viability of *KmKU70*^+^ and *KmKU70*^−^ strains expressing *Rsa*I cultured in liquid YPGal with vigorous shaking. Ku70 is required to restore viability after DSB induction in *Km*. Results from three biological replicates are plotted on the graph, with the error bars representing the standard deviation of the means. Bars with different lowercase letters are significantly different at a 0.05 level of significance (*p* < 0.05).

The yeast *K. marxianus* exhibits efficient non-homologous end-joining (NHEJ) activity to repair DNA double-strand breaks [33,34,35]. To gain further insight into changes in chromosomal integrity during DNA break repair in this yeast, we constructed strains expressing the *Rsa*I gene under the control of the galactose-inducible promoter P*_GAL10_*. In a static culture, the *KmKU70*^+^ (*Rsa*I-transformant) strain showed growth defects on inducible YPGal medium (Figure 1A). After an initial drop at 3 h of culture in liquid YPGal, the *KmKU70*^+^ strain expressing the *Rsa*I gene stabilized and began to show a trend of recovery. In comparison, the *KmKU70*^−^ strain continued to decline with longer incubation time (Figure 1B). The difference between the *KmKU70*^+^ and *KmKU70*^−^ strains is statistically significant (*p* < 0.05; *n* = 3) at 9 and 12 h, indicating that the *KmKU70*^+^ strain expressing the *Rsa*I endonuclease could recover its growth after rearranging the chromosomes into properly functioning structures.

### 3.2. Effects of Induction of RsaI in KU70^+^ and KU70^−^ Cells

**Figure 2:**
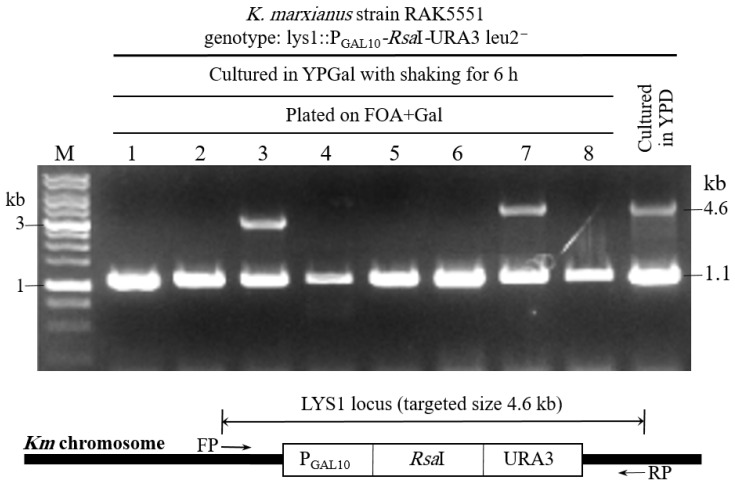
Fate of the targeted disruption of the *LYS1* locus upon the induction of the restriction enzyme *Rsa*I. The *KmKU70*^+^ viable strain (RAK5551) was cultured in YPGal for 6 h with vigorous shaking and plated on FOA+Gal. Eight colonies were tested by means of PCR for the targeted construct (4.6 kb). PCR results showed that six of the eight strains had lost the construct at the *LYS1* locus, namely, RAK6480 (lane 1), RAK6481 (lane 2), RAK6483 (lane 4), RAK6484 (lane 5), RAK6485 (lane 6), and RAK6487 (lane 8). Two strains, RAK6482 (lane 3) and RAK6486 (lane 7), retained the transformed construct in the *LYS1* locus. The DNA band at 1.1 kb corresponds to the *Km ACT1* gene and was used as a reference for DNA loading (see Section 2 for details). A schematic depiction of the gene construct targeted at the *LYS1* locus is shown at the bottom of the agarose gel. M, DNA molecular weight marker (1 kb DNA Ladder, Sib enzyme); FP, forward primer; RP, reverse primer.

**Figure 3:**
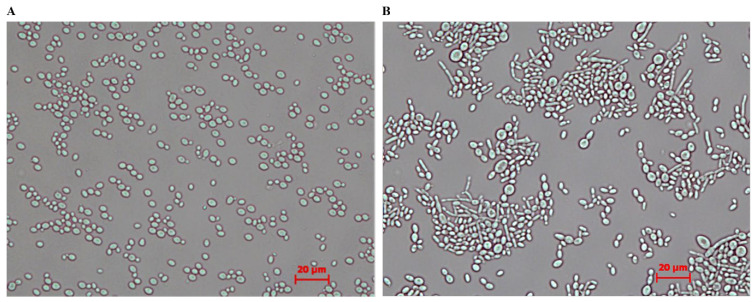
Morphological alteration of *KmKU70*^+^ cells expressing *Rsa*I. (**A**) *K. marxianus* DMKU3-1042 and (**B**) *Rsa*I-transformed *K. marxianus* strains were cultured in YPGal, grown at 30 °C for 24 h, and examined under a microscope. *K. marxianus* DMKU3-1042 cells exhibited normal growth, whereas the *Rsa*I-transformed cells showed deformed morphological cell structures. Scale bar = 20 μm.

The *Km LYS1* locus is located on chromosome 4 (NCBI reference sequence: NC_036028.1) according to the genome sequence of the strain DMKU3-1042, and its sequence coordinates range from nucleotides 856,079 to 857,197, encoding 372 amino acids [55]. The strain *KmKU70*^+^ expressing *Rsa*I (RAK5551) was incubated in a YPGal liquid medium for 6 h with vigorous shaking. The induced cells were plated on FOA-containing galactose (FOA+Gal medium). The colonies that lost the *Rsa*I were expected to grow on the FOA+Gal medium. Colonies that show similar growth to the *KU70*^+^ cells in the FOA+Gal solid medium may have undergone mutation in *Rsa*I, partial deletion in the transformed construct, or the entire construct may have been deleted through the action of the restriction enzyme *Rsa*I. Eight colonies (Table 1) were tested by means of PCR for the presence or absence of the inserted fusion construct (P*GAL10*-*Rsa*I-*URA3*). Only two colonies (RAK6482 and RAK6486) retained the *Rsa*I expression cassette at the *Km LYS1* locus; the remaining six colonies had lost the cassette from the *LYS1* locus (Figure 2), suggesting a structural change in the chromosomes. The *KmKU70*^+^ yeast cells expressing *Rsa*I (RAK5551) grown in YPGal exhibited morphological alterations under a microscope (Figure 3). Some cells displayed rod-shaped structures, whereas others exhibited elongated structures compared to the wild-type *Km* cells. Our results suggest that some of the elongated cells were attached, forming long threads with distinct invaginations.

### 3.3. Pulsed-Field Gel Electrophoresis (PFGE) of K. marxianus Expressing RsaI Recovered on FOA-Gal

**Figure 4:**
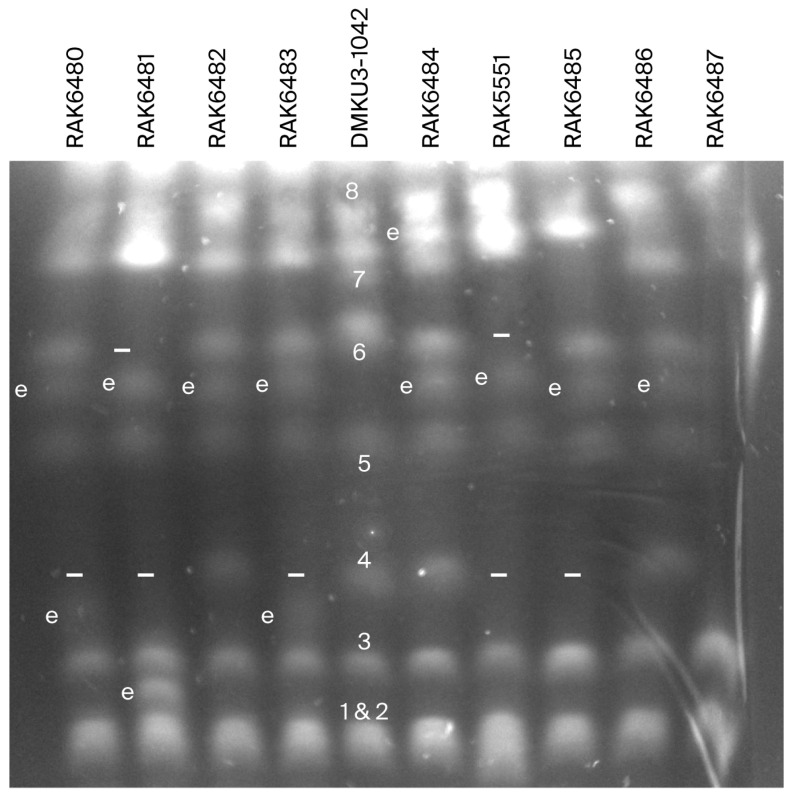
Chromosomal DNA separation on pulsed-field gel electrophoresis (PFGE). PFGE of *K. marxianus* chromosomes extracted from nine viable *Rsa*I transformants, which had undergone chromosome rearrangement in addition to the chromosomes from the strain DMKU3-1042. The electrophoresis gel contains chromosomes from the strains RAK6480 (lane 1), RAK6481 (lane 2), RAK6482 (lane 3), RAK6483 (lane 4), DMKU3-1042 (the wild type) (lane 5), RAK6484 (lane 6), RAK5551 (lane 7), RAK6485 (lane 8). Numbers on the gel indicate the chromosomes from DMKU3-1042, whereas the distinct extra bands from the *Rsa*I-expressing strains are marked by the letter (e), and the chromosomes that have disappeared are indicated by the (−) sign. A summary of new bands or missing chromosomes from each strain relative to DMKU3-1042 is presented in Table 2.

Chromosomes from the *KmKU70*^+^ colonies expressing *Rsa*I depicted in Figure 2 were separated by means of PFGE (Figure 4). PFGE-separated chromosomes of the strain DMKU3-1042 (lane 5) were indicated by numeric labels on the gel. Chromosomes 1 and 2 migrated together as a dense band at the bottom of the gel, and therefore, they are referred to as chr1 and 2. An apparent variation was discovered in the chromosomal size of some *Rsa*I-expressing strains, as evidenced by their separation on the gel. We detected an extra band (highlighted as e on the gel panel) from the strains RAK6480 (lane 1), RAK6481 (lane 2), RAK6482 (lane 3), RAK6483 (lane 4), RAK6484 (lane 6), RAK5551 (lane 7), RAK6485 (lane 8), and RAK6486 (lane 9). Its position lies between chr5 and chr6 in DMKU3-1042 (lane 5). The strain RAK6481 possesses an additional band between chr1 and 2 and chromosome 3 compared to DMKU3-1042. The strains RAK6480 and RAK6483 each contain an extra band above chr3 of DMKU3-1042, which is undetectable in the other strains. Moreover, the strain RAK6484 contains an additional band located between chromosomes 7 and 8 of DMKU3-1042. Chromosome 4, which includes the targeted *LYS1* locus, disappeared from the strains RAK6480, RAK6481, RAK6483, RAK5551, and RAK6485 (its position is highlighted by a − sign on the gel panel). Chromosome 6 of DMKU3-1042 appears denser and relatively higher on the gel than the relative bands from the strains RAK6480, RAK6482, RAK6483, RAK6484, RAK6485, and RAK6486. Larger chromosomes of the strain RAK5551 densely migrated to the top of the gel and exhibited incomplete separation. Chromosomes 1 and 2, in addition to chromosome 3 of DMKU3-1042, appeared in all tested strains. A noticeable difference was detected in the larger chromosomes at the top of the gel between the strains RAK6481, RAK6482, RAK6483, RAK6484, RAK5551, RAK6485, and RAK6486 compared to DMKU3-1042. Most bands of the strain RAK6487 (lane 10), except for two at the bottom, were distorted during migration on the gel, rendering them indistinguishable. A consistent chromosomal change was the introduction of an extra chromosome between chromosomes 5 and 6 of the wild-type strain, in addition to the loss of chromosome 4 in the majority of tested strains (Table 2; Figure 4). The PFGE result is consistent with the PCR data, as the PFGE procedure confirmed the presence of chromosome 4, and PCR results showed retention of the transformed construct at the *LYS1* locus in strains RAK6482 and RAK6486. As all tested strains are viable on FOA-Gal and exhibit discrete chromosomal migration on the gel, the yeast strains underwent significant karyotype engineering, resulting in rearranged or aberrant chromosomes.

## 4. Discussion

An in vivo genetic assay was developed to study non-conservative (i.e., leading to some changes in the DNA sequence) intra-chromosomal deletions at regions of non-tandem direct repeats in *Schizosaccharomyces pombe* [20]. This chromosomal rearrangement may lead to local species adaptations and diversification [56] if it is not lethal. Researchers have demonstrated that yeast chromosomal structural rearrangements have successfully enhanced industrially important phenotypes, including the production of novel medicines, nutritional supplements, anti-tumor molecules, and tolerance to environmental stress and drug resistance [24,57].

In this study, we aimed to determine whether inducing DNA DSBs affected the chromosomal integrity of *K. marxianus*. The restriction endonuclease *Rsa*I recognizes the sequence GTAC, cleaves it between T and A, and produces blunt ends. Draft genome sequences of *K. marxianus* strains KCTC 17555 [58], DMB1 [59], and CCT 7735 [60], in addition to complete genome sequences of the strains DMKU3-1042 [55] and NBRC 1777 [61], have been identified. The strains DMKU3-1042 and NBRC 1777 each possess eight chromosomes in their genomes [55,61]. The sequences of the smallest chromosomes, 7 and 8, are 963,718 and 939,718 bp, respectively (Supplementary Table S1). Analysis of the genome of strain DMKU3-1042 using the software SnapGene© version 8.2 revealed 5009, 5139, 4633, 4236, 3986, 3599, 2870, and 3995 *Rsa*I recognition sites on chromosomes 1, 2, 3, 4, 5, 6, 7, and 8, respectively, in addition to 42 recognition sites in the mitochondrial DNA (Supplementary Table S1). In total, roughly 33,499 recognition sites for the restriction enzyme *Rsa*I were found in the genome of *K. marxianus*. It has been reported that *K. marxianus* strain 4G5 can switch its mating types [62]. Haploid cells may become diploids and produce spores in response to harsh environmental conditions [62]. Nevertheless, the strain DMKU3-1042 employed in this study is haploid [32]. The expression of the restriction enzyme *Rsa*I caused a growth defect in the *KmKu70*^+^ strain grown on solid medium (Figure 1A). The lethality of *Rsa*I in the *KmKU70*^−^ strain is likely due to the loss of the NHEJ machinery that repairs broken DNA. This finding is consistent with reports demonstrating that cells employ various strategies to escape selective pressure [63]. To escape *CRISPR/Cas*-mediated knockouts, cells undergo target-site mutations, such as nucleotide substitutions or insertions/deletions. These mutations prevent guide RNA binding or *Cas*9 cleavage, in addition to alternative splicing or exon skipping, leading to truncated proteins that retain partial function [63]. Multiplex genome editing in yeast using CRISPR/*Cas*9 [64] enabled simultaneous targeting of multiple genes, facilitating the reconstruction of complex biosynthetic pathways. By employing multiple guide RNAs, the platform can induce various mutations across different loci, enabling comprehensive genetic modifications and the study of gene interactions in metabolic engineering applications [64]. Adaptive evolution of *K. marxianus* toward tolerance of high ethanol concentrations was demonstrated during adaptive laboratory evolution [65].

A notable observation was made regarding the morphology of induced *Rsa*I-expressing *Km* strains. Microscopic examination revealed that some cells appeared rod-shaped, whereas others were elongated compared to wild-type *Km* cells (Figure 3). Some elongated cells were attached, forming long threads with distinct invaginations. It has been reported that when cell damage occurs, the cell cycle checkpoints halt or delay the cycle to enable the cell to ameliorate the damage before division. Elongated or large-budded yeast cells with undivided nuclei are mostly indicative of the G2/M checkpoint, which monitors DNA damage before mitosis [66,67]. The morphological appearance of *Rsa*I-expressing *Km* survivors is primarily due to a cell cycle delay in DNA repair or due to oxidative stress [68]. However, the *Rsa*I-transformed *KmKu70*^+^ strain recovered its growth when evaluated in liquid culture with vigorous shaking (Figure 1B), which likely enhanced the selective pressure for cells to escape *Rsa*I expression by mutations in the expression cassette or loss of the entire *LYS1* locus. The difference in growth between *KmKU70*^+^ and *KmKU70*^−^ *Rsa*I-transformed strains suggests that NHEJ function is necessary for recovery after DSB. The authors of a study involving a *rad52* mutant *S. cerevisiae* strain, which is deficient in DSB repair, expressing the *Eco*RI endonuclease that produces four-base-pair cohesive ends, found that the restriction enzyme’s lethality was exacerbated under induction conditions [69].

FOA selection is enriched for cells that have lost the selection marker *URA3* through mutation or deletion of the *Rsa*I expression cassette. Growth of *Rsa*I-expressing *Km* strains on FOA-Gal suggested that the viable cells may have undergone mutation, partial deletion, and/or deletion of the entire targeted region. The latter was confirmed by means of colony PCR (Figure 2). Six viable colonies revealed the loss of the *LYS1* gene from its original locus. For further evaluation, the chromosomes of these strains were visualized by means of PFGE, a widely used technique for karyotyping yeast and human genomes [24,70,71,72]. In a previous study on chromosome polymorphisms in Italian *K. marxianus* cheese strains, the results revealed seven PFGE patterns, differing in chromosome size and number [73], and showed that the number of visualized chromosomal bands ranged from four to seven, with sizes of roughly 1.0 to 2.7 Mb [73]. In our study, six to seven bands of chromosomes from the wild-type *Km* strain DMKU3-1042 were resolved via PFGE. Conversely, the sequence data of the same strain revealed eight chromosomes in its genome [55]. This discrepancy may be due to the joint migration of closely sized chromosomes on the gel. PFGE of the chromosomes from the induced *Rsa*I-expressing *Km* strains showed distinct variations (Figure 4). While *Rsa*I sites are abundant in the genome of *Km* DMKU3-1042, selection pressure at the targeted *LYS1* locus and survival bias likely dominate observed karyotypes, rather than random genome-wide cleavage. The strains RAK6482 and RAK6486, which retained the expression cassette at the *LYS1* locus (Figure 2), showed a similar pattern of chromosomal migration on the PFGE. They also retained chr4, similar to the DMKU3-1042 strain. However, the band corresponding to chromosome 4 was not detected in the strains RAK6481, RAK6485, RAK6487, and RAK5551. In contrast, new bands of different sizes appeared in the other induced strains. Taken together, these data confirm the structural rearrangement in the chromosomes of induced *Rsa*I-expressing *K. marxianus*, resulting in new strains with chromosomal migration on the gel that differs from that of their ancestor strain, DMKU3-1042. Strains harboring the active NHEJ pathway utilize this machinery to restore the integrity of their chromosomes. Further investigation is needed to characterize the molecular nature of karyotype rearrangements inferred from PFGE and the role of HR as a potential backup repair pathway in the *KmKU70*^+^ strain.

## 5. Conclusions

Our study’s findings demonstrate that the *ku70* mutant strain of *K. marxianus* expressing the endonuclease *Rsa*I generates multiple chromosomal fragments. Despite the presence of approximately 33,599 *Rsa*I recognition sites in the yeast genome, some strains with newly rearranged chromosomes can survive. These strains evade the endonuclease’s lethality by rearranging their chromosomes in various patterns, suggesting a powerful random genome-shuffling technique for *engineering K. marxianus strains*. Our results highlight the potency of the NHEJ repair pathway in maintaining genome stability through chromosomal rearrangements and aid in directing future studies toward genome engineering and adaptive evolution strategies in the thermotolerant yeast *K. marxianus* DMKU3-1042.

## Figures and Tables

**Table 1:** Strains used in this study.

Strain *	Genotype	Source
*K. marxianus*		
DMKU3-1042	Wild-type (WT)	[41]
RAK3605	*ura3*	[32]
RAK3627	*ura3 lys1::ScURA3*	[32,34]
RAK4174	*ura3 leu2*	[42]
RAK4736	*ura3 ku70* *Δ* *::ScLEU2*	[32]
RAK5332	*ku70Δ::ScLEU2 lys1*::P*_GAL10_-Rsa*I*-URA3*	RAK4736 transformant (This study)
RAK5551	*lys1*::P*_GAL10_-Rsa*I*-URA3 leu2*	RAK4174 transformant (This study)
RAK6480	*lys1*::P*_GAL10_-Rsa*I*-URA3 leu2*	YPGal-induced RAK5551
RAK6481	*lys1*::P*_GAL10_-Rsa*I*-URA3 leu2*	YPGal-induced RAK5551
RAK6482	*lys1*::P*_GAL10_-Rsa*I*-URA3 leu2*	YPGal-induced RAK5551
RAK6483	*lys1*::P*_GAL10_-Rsa*I*-URA3 leu2*	YPGal-induced RAK5551
RAK6484	*lys1*::P*_GAL10_-Rsa*I*-URA3 leu2*	YPGal-induced RAK5551
RAK6485	*lys1*::P*_GAL10_-Rsa*I*-URA3 leu2*	YPGal-induced RAK5551
RAK6486	*lys1*::P*_GAL10_-Rsa*I*-URA3 leu2*	YPGal-induced RAK5551
RAK6487	*lys1*::P*_GAL10_-Rsa*I*-URA3 leu2*	YPGal-induced RAK5551
*S. cerevisiae*		
BY4704	*MATa ade2Δ::hisG his3Δ200 leu2Δ0 lys2Δ0 met15Δ0 trp1Δ63*	[43]
BY4743	*MATa/alpha his3Δ1/his3Δ1 leu2Δ0/leu2Δ0 met15Δ0/MET15 LYS2/lys2Δ0 ura3Δ0/ura3Δ0*	[40,43]
RAK3940	*MATa his3Δ200 leu2Δ0 met15Δ0 trp1Δ63 ura3Δ0::P_GAL10_-yEGFP-15C-PpHIS3*	[40,44]
RAK5331	*MATa his3Δ200 leu2Δ0 met15Δ0 trp1Δ63 ura3Δ0::P_GAL10_-RsaⅠ-15C-URA3*	This study

* The strains from RAK6480 to RAK6487 were recovered after the galactose induction of the strain RAK5551. Only the strains RAK6482 and RAK6486 retained the entire transformed construct. The other six strains lost the targeted construct into the *LYS1* locus.

**Table 2:** Major karyotype engineering observed in *Rsa*I-transformed strains relative to the wild-type *K. marxianus* DMKU3-1042 using PFGE.

DMKU3-1042 Chromosomes	RAK6480 Band *	RAK6481 Band	RAK6482 Band	RAK6483 Band	RAK6484 Band	RAK5551 Band	RAK6485 Band	RAK6486 Band	RAK6487 Band
1 & 2	1 & 2	1 & 2	1 & 2	1 & 2	1 & 2	1 & 2	1 & 2	1 & 2	1 & 2
		New							
3	3	3	3	3	3	3	3	3	3
	New			New					
4	Missing	Missing	4	Missing	4	Missing	Missing	4	Missing
5	5	5	5	5	5	5	5	5	Distorted
	New	New	New	New	New	New	New	New	
6		Missing				Missing			Distorted
7	7	7	7	7	7	7	7	7	7
					New	New			
8	8	8	8	8	8	8	8	8	8

* Chromosomal bands are tabulated from Figure 4.

## Data Availability

The datasets generated and/or analyzed during the current study are available from the corresponding author upon reasonable request.

## Supplementary Materials

The following supporting information can be downloaded at https://www.mdpi.com/article/10.3390/cimb48030252/s1, Figure S1: Complete sequence of the fusion construct with annotations; Figure S2: Full-length electrophoresis gel image for Figure 2; Figure S3: Full-length electrophoresis gel image for Figure 4; Table S1: Recognition sites for the restriction enzyme *Rsa*I in the genome of *Kluyveromyces marxianus* strain DMKU3-1042.
