# Supplementary material for: Expression of Endonuclease RsaI Induces Chromosomal Rearrangement in the Yeast Kluyveromyces marxianus
Source: Curr Issues Mol Biol. 2026 Feb 26;48(3):252. doi: 10.3390/cimb48030252 (PMC13025109; doi:10.3390/cimb48030252)
Supplement: Supplementary file 1 [file cimb-48-00252-s001.zip › cimb-4158721-supplementary.pdf]

Forward Primer (FP)

AATGGCTCCCACATTGCTGTTGGGTTATTGACAAAGCTTCTAATATTCAAGACAGAAACGGCTA
AGGTACATAGAGAAATCGATTTTGGTAGGAAGACTGTGATGCATCTTGAGAGAGAAGTTATTGA
TGGGGAGGAATATCTAGTTGTTGGGTTGAACGACGGTAGTGTCCAATTTTGGAAAACATCGAAA
CTTTATGAGGATGTACCAGAACCCGAAGTCGAAGAAGAAGAAGAAGAAGAAGAAGAAGAAGAGG
AGGCTAATGACGATAAGGAAAAAAGAAGCTAAAACCCCATCTTTAGAGCCTGACTTTACCTTAC
TAGGTCACAGCAATCGTGTTAAAGACTTCCAATTTTACAAAAACGAGCATGGTCATTATTTAGT
CACTATTGGATCAGATGGTAGAATTGTTGTTTGGGACATGAAGATTAAGGACCAAATCGCTGTC
TATGATTCCGGTGAAAGATTGAATTGTGTCGCCGTATGTGACGAAAGTATTGAAAAATATGACA
CTGTTAAGAAGAGAACATCAGAAGACGTAGAATTAGGAGAACAAAGTGAAGCTGAGGCAGATCC
AGAAGAATTGAAGAAGATTATGATGGGCAAAACATCTAAAAGCAAGAAAAAGAAGGGTTCAAAA
AGAGAAAGGTGGACATTCAATTGGAGTAACTTATTTAGGCATATCAATATATATATATATATAT
ATATACATTGTTAGGTACTAACAAAAAGTACTTATTGTACAATAAAATTAAACAGCGTTTAGTC
GATTATCTTCCTGAAGAATAATTTATGCCGACTTACTGCTTGAAAGTGAAAGTATTTTGGAGCA
TTTTTTAAGCAACTATAGCTTTGAAGTCCTTCCAACCTTAGCGGAGAATTTCTCATAGAGCTTT
CTTGCCTTGACCCAAACAGAAGCTGTATCTCTTTGGGGAAGTTGTTCCAAGGAAGGCAAGAGAT
CACGAGAGAATGACTCTGAGGCCTCTTTTGGAAGCAATGA**CCCCCGGGGGCCCCC**GGAGGGCTG
TCGCCCGCTCGGCGGCTTCTAATCCGTACTTCAATATAGCAATGAGCAGTTAAGCGTATTACTG
AAAGTTCCAAAGAGAAGGTTTTTTAGGCTAAGATAATGGGGCTCTTTACATTTCCACAACATA
TAAGTAAGATTAGATATGGATATGTATATGGTGGTAATGCCATGTAATATGATTATTAAACTTC
TTTGCGTCCATCCAAAAAAAAAGTAAGAATTTTTGAAAATTCAATATAAATCCAAAAAAAAAGT
AAGAATTTTTGAAAATTCAATATAAatggagcgccgcttccagctgcgctgggacgaggaggag
cTGGCCCGCGCCTTCAAGGTGACCACCAAGGACGTGCGCGAgtacctgaccgacggccgccgcg
tgagcttcatcatcgagCGCCGCCTGATGTGGGAGAACCCCGGCTGGAAGCTGGCCCccagcga
gggcgccggctacgacctgctggaccccgagggCGGCATGTGGGAGGTGCGCAGCATCACCCGC
CAGGGCGTGtacttcaaccccagcaaccaggtgggcagcggccgcaagtTCAACGAGGACGGCT
TCCAGCTGAAGATGAGCGGCATCAAgggcttcatcctgagcgacatcgtgggcttcccсctggt
gGACGTGTACGTGGTGCCCGTGGAGAACGTGCTGCGCTGGCaccaggcccgcgccctgggcgcc
aacgccaaggtgagccgCGAGAAGTTCCTGCGCGACATGGTGCGCGACATCCGCCACTAA**CCCC**
**CCCCCCCCCCC**GAAGAGTATTGAGAAGGGCAACGGTTCATCATCTCATGGATCTGCACATGAAC
AAACACCAGAGTCAAACGACGTTGAAATTGAGGCTACTGCGCCAATTGATGACAATACAGACGA
TGATAACAAACCGAAGTTATCTGATGTAGAAAAGGATTAAAGATGCTAAGAGATAGTGATGATA
TTTCATAAATAATGTAATTCTATATATGTTAATTACCTTTTTTGCGAGGCATATTTATGGTGAA
GGATAAGTTTTGACCATCAAAGAAGGTTAATGTGGCTGTGGTTTCAGGGTCCATAAAGCTTTTC
AATTCATCTTTTTTTTTTTTGTTCTTTTTTTTGATTCCGGTTTCTTTGAAATTTTTTTGATTCG
GTAATCTCCGAGCAGAAGGAAGAACGAAGGAAGGAGCACAGACTTAGATTGGTATATATACGCA
TATGTGGTGTTGAAGAAACATGAAATTGCCCAGTATTCTTAACCCAACTGCACAGAACAAAAAC
CTGCAGGAAACGAAGATAAATCATGTCGAAAGCTACATATAAGGAACGTGCTGCTACTCATCCT
AGTCCTGTTGCTGCCAAGCTATTTAATATCATGCACGAAAAGCAAACAAACTTGTGTGCTTCAT
TGGATGTTCGTACCACCAAGGAATTACTGGAGTTAGTTGAAGCATTAGGTCCCAAAATTTGTTT
ACTAAAAACACATGTGGATATCTTGACTGATTTTTCCATGGAGGGCACAGTTAAGCCGCTAAAG
GCATTATCCGCCAAGTACAATTTTTTACTCTTCGAAGACAGAAAATTTGCTGACATTGGTAATA

CAGTCAAATTGCAGTACTCTGCGGGTGTATACAGAATAGCAGAATGGGCAGACATTACGAATGC
ACACGGTGTGGTGGGCCCAGGTATTGTTAGCGGTTTGAAGCAGGCGGCGGAAGAAGTAACAAAG
GAACCTAGAGGCCTTTTGATGTTAGCAGAATTGTCATGCAAGGGCTCCCTAGCTACTGGAGAAT
ATACTAAGGGTACTGTTGACATTGCGAAGAGCGACAAAGATTTTGTTATCGGCTTTATTGCTCA
AAGAGACATGGGTGGAAGAGATGAAGGTTACGATTGGTTGATTATGACACCCGGTGTGGGTTTA
GATGACAAGGGAGACGCATTGGGTCAACAGTATAGAACCGTGGATGATGTGGTCTCTACAGGAT
CTGACATTATTATTGTTGGAAGAGGACTATTTGCAAAGGGAAGGGATGCTAAGGTAGAGGGTGA
ACGTTACAGAAAAGCAGGCTGGGAAGCATATTTGAGAAGATGCGGCCAGCAAAACTAAAAAACT
GTATTATAAGTAAATGCATGTATACTAAACTCACAAATTAGAGCTTCAATTTAATTATATCAGT
TATTACCCGGAATCTCGGTCGTAATGATTTCTATAATGACGAAAAAAAAAAAAATTGGAAAGAA
AAAGCTTCATGGCCTTTATAAAAAGGAACTATCCAATACCTCGCCAGAACCAAGTAACAGTATT
TTACGGGGCACAAATCAAGAACAATAAGACAGGACTGTAAAGATGGACGCATTGAACTCCAAAG
AACAACAAGAGTTCCAAAAAGTAGTGGAACAAAAGCAAATGAAGGATTTCATGCGTTTGTACTC
TAATCTGGTAGAAAGATGTTTCACAGACTGTGTCAATGACTTCACAACATGGCAAATGATCAAT
TGAACACACTGAAAGTTTTGGACCTGCAGTTGTTGGAACCACTACTGTTGGAGCATCGAATGTT
GTATTTATAGAGTAAATTGGAACGGGATTGTGAGGGTTTGTTGTGTCTGCAGAAACATCGACAA
CAGTTCTCAACCTTCTGTTAGGATTATTCAAAAGCTTGTTGTTAATGAATGGGGGAATTGGCTT
GGATAAGTAGATACAGTTGATGAATATGTCTGCATCTGCGATTTCTTGAAATGGCCCCCCGCGA
GCAGTTTCCTTCATATCCCACTTGATAATATTCTCCTCCGGCAAACCGCACTTGCGCAACAAGT
CAACAGCACCGGAACCACATCTACCTAATGCACCAATAATCAATACCTTAGGCTTTCTAGCTCC
TTTCTTCAAAGCTTCCTGGTAATCTCTACGAACATCCTTGATCATGGCAGTCTCATTTTCGTAA
GGTTCGATGGCGCCCAAATTCTCAGAGTCAGCATGAGTTTGCTTGAAAGCCCAGTCCCTGATAC
CCACTGCAGCACCAGCAAACCCAGCGTAAAACCCGAAGGCTGCTACTCTTCTGCCTTGGTCGTT
TTCCAAGAACTCAAGGTCATATAACACACCATTCCCGTTCTTAAAACGACGCAAGACGTCCTCC
CAACCAGCCTGGTTCTTGTAACAATGGGCAAATTGAATATGCTCATGAACTAGAGGGAAAGTGT
CCGTTTCTGGCATTTCTTTCAACCCAATAATAATTCTATCACGTGGAGCAGTCTTCCATGATCC
TGTTGGGACAATAATGGCTCCAACCTTTCTGTAATCTTCAATATCAAACGCGGATTGTGCGCTT
TCTTCAACATACACTTTGAAACCCTTCTCCAGCAACTTCTTAGTAGTTGTTGGAGTTAAAGCGG
TACGTGCTTCCAATGGCTTAGTTTCAGCTCTCAAATGAAGTGTCACAGTCATGTTTGTATTATT
TCGGTAGTACTAGATATATCAATCT-**4505**

Reverse primer (RP)

**Figure S1**. Sequence of the fusion construct [***KmLYS1***-**P*GAL10***-***Rsa*I**-***URA3***-***KmLYS1***] targeted into the *LYS1* locus of the yeast *K. marxianus*. Sequences in green are for *KmLYS1*, in orange are for *GAL10* promoter, in brown are for the restriction endonuclease *Rsa*I, in blue are for *URA3*, and in black are the fusion sequences. The *Rsa*I recognition sites are in red.

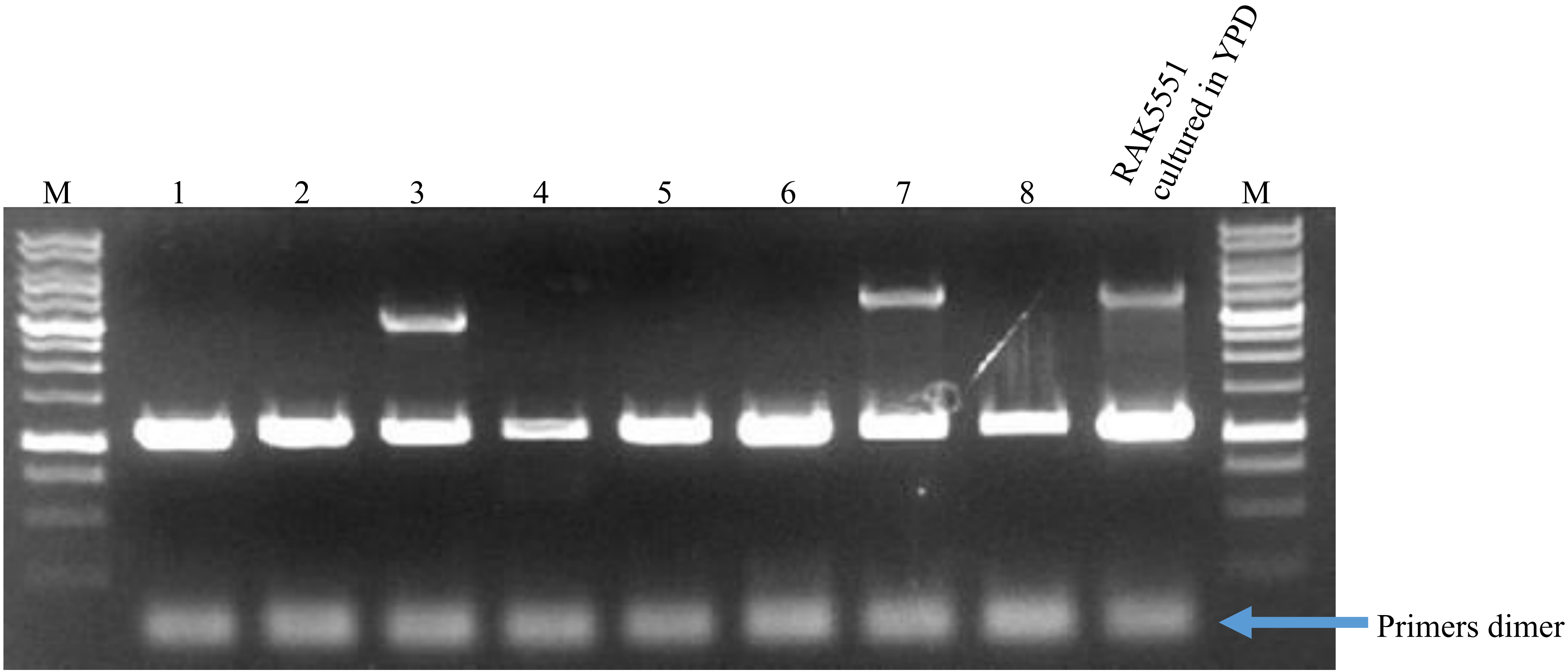


**Figure S2**. Full-length electrophoresis gel image for **Figure 2.** For details, see legends for Figure 2

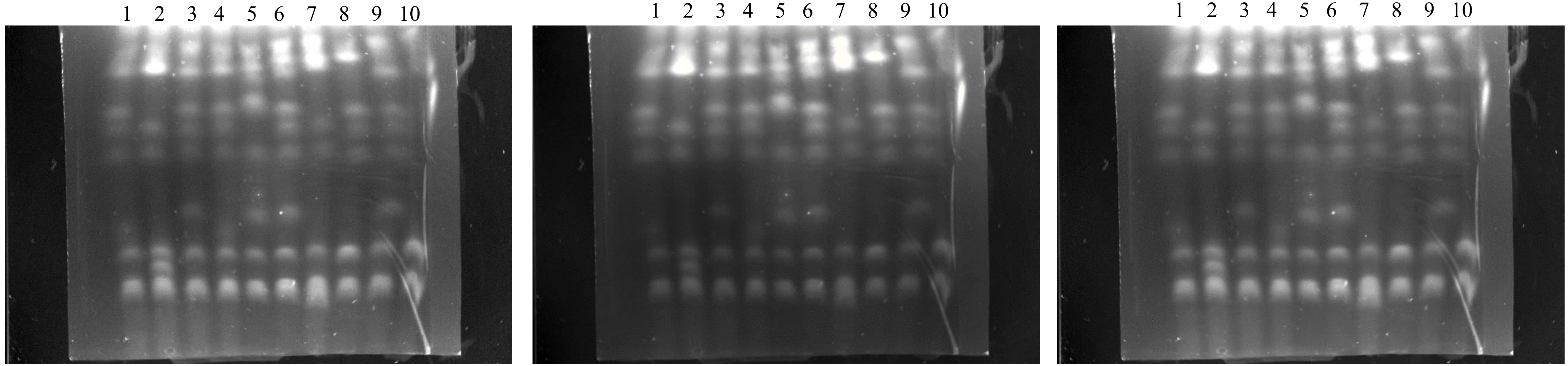


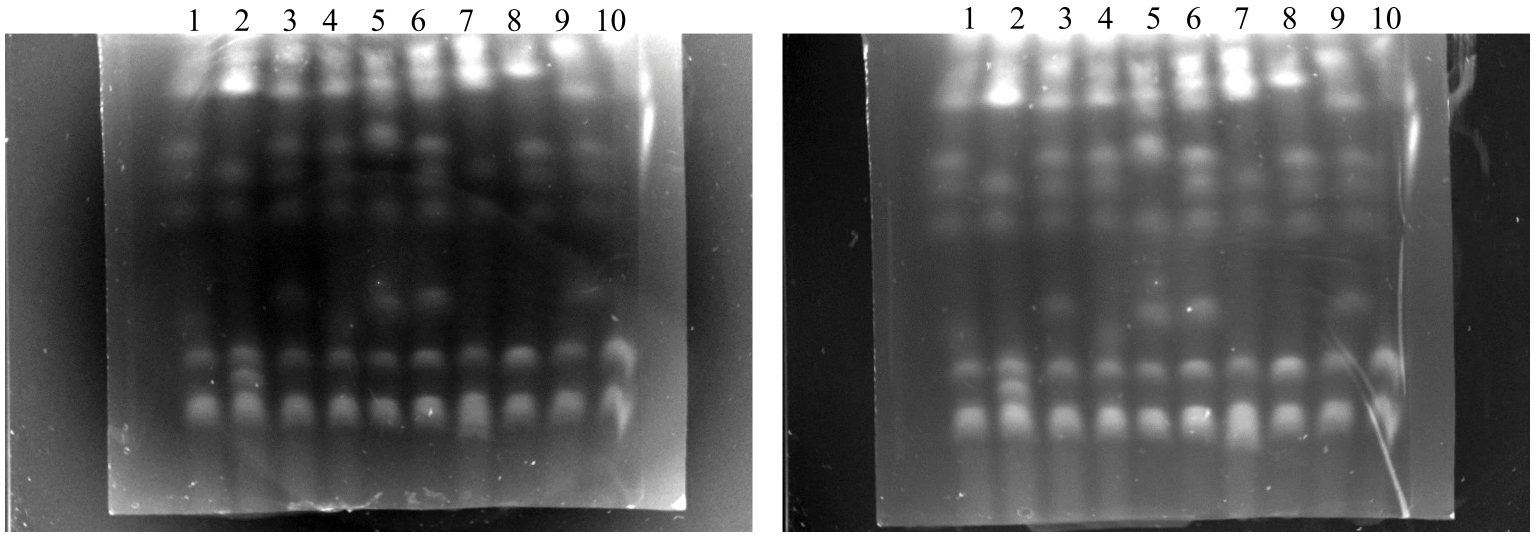


**Lane**:
1. RAK6480
2. RAK6481
3. RAK6482
4. RAK6483
5. *K. marxianus* DMKU3-1042 (WT)
6. RAK6484
7. RAK5551
8. RAK6485
9. RAK6486
10. RAk6487

**Figure S3.** Full-length electrophoresis gel image for **Figure 4.** The electrophoresis gel was stained with ethidium bromide and exposed to varying exposure times. For details, refer to the legends for Fig. 4.

**Supplementary Table S1**. Recognition sites for the restriction enzyme *Rsa*I in the genome of *Kluyveromyces marxianus* strain DMKU3-1042.

| *Km* chromosome # | Size (bp) | Accession # | *Rsa*I* recognition sites |
|---|---|---|---|
| 1 | 1,745,387 | NC_036025.1 | 5,009 |
| 2 | 1,711,476 | NC_036026.1 | 5,139 |
| 3 | 1,588,169 | NC_036027.1 | 4,633 |
| 4 | 1,421,472 | NC_036028.1 | 4,236 |
| 5 | 1,353,011 | NC_036029.1 | 3,986 |
| 6 | 1,197,921 | NC_036030.1 | 3,599 |
| 7 | 963,005 | NC_036031.1 | 2,870 |
| 8 | 939,718 | NC_036032.1 | 3,995 |
| Mitochondrial DNA | 46,308 | NC_036023.1 | 42 |
| **Total** | **10,966,467** | | **33,509** |

*The recognition sequence for *Rsa*I: 5′–GT|AC–3′.

**Reference:**

Lertwattanasakul N, Kosaka T, Hosoyama A, Suzuki Y, Rodrussamee N, Matsutani M, Murata M, Fujimoto N, Suprayogi, Tsuchikane K, Limtong S, Fujita N, Yamada M (2015) Genetic basis of the highly efficient yeast *Kluyveromyces marxianus*: complete genome sequence and transcriptome analyses. Biotechnol Biofuels 8:47. https://doi.org/10.1186/s13068-015-0227-x.
